# Editorial

**Published:** 1846-07

**Authors:** 


THE MEDICAL EXAMINER.

PHILADELPHIA, JULY, 1846.

### ALTERATIONS OF NAMES.

It is amusing to witness the mutations which proper names experience in going the rounds of the journals. In "Braithwaite's Retrospect," for example, we have an article on the use of Creosote in Ophthalmia, &c., by *Dr. Zanesville*, who, we suppose, is *Dr. Hildreth of Zanesville;* his communication doubtless passed into the French journals, and from them into the English, with the name thus strangely *estropiée.* An analogous case we have in that of Dr. Perry of Philadelphia, who wrote an article for Dunglison's "American Intelligencer," in 1838, on the use of Chloride of Silver in various diseases. This article was extensively noticed, as every curative agent is sure to be, which is brought forward with high encomiums, no matter whether well or ill founded, and amongst others in the French journals, where Dr. Perry's name underwent the slight change into *M. Perri;* and as *M. Perri*, it is cited back in one of our standard works. Again, in the "*Annuaire de Therapeutique, of Bouchardat,*" for 1845, there is the following caption to an article: "*Colchicum: Remarks upon its employment against the gout,* (*Bentley.*) After which we are told that *M. Bentley Tood* has inserted in a treatise on the gout several remarks worthy of attention in the employment of colchicum,"—both *M. Bentley*, and *M. Bentley Tood*, being *Dr. Bentley Todd!*

These ridiculous and inexcusable errors are occurring constantly; but far more frequently with our Gallic brethren than with others.

### HIPPOCRATES AND GALEN.

Some time since, we mentioned that the writings of these eminent fathers in medicine were about being rendered into English, by the venerable John Redman Coxe, M. D. We now have the pleasure of announcing that the work is in press, and will be published, "epitomized and rendered into English," in one large octavo of from seven to eight hundred pages, early in August next.

We venture the prediction that, when the volume appears, it will be found that these ancient practitioners were familiar with many things now deemed *new discoveries*, by some, too, who are not classed with superficial readers. The great learning and accuracy of the translator, affords a sure guarantee that the work as it comes to us will be a faithful representation of the originals.

---

ANNUAL CONVENTION OF THE CONNECTICUT STATE MEDICAL SOCIETY.

In the State of Connecticut, the members of the medical profession are organized into County Societies, and these again constitute a State Society, which holds annual conventions. The assemblage of so large a number of the profession is always fruitful of interest, and when their proceedings have the force of law, as in our sister state, they become all the more important.

The Convention for the present year was held at New Haven on the 13th and 14th of May last. From the published proceedings, for a copy of which we are indebted to the Secretary, Dr. Russell, we learn that the following gentlemen were elected officers, viz:

Archibald Welsh, M. D., President.
Dyer T. Brainard, M. D., Vice President.
V. M. Dow, M. D., Treasurer.
Gurdon W. Russell, M. D., Secretary.

At the Convention a number of gentlemen were elected Fellows of the Society, and various matters transacted. Among other things, the following resolution was adopted, viz:

"Resolved, That the county meetings have power to appoint one delegate each to the National Medical Convention, to be holden in Philadelphia, on the first Wednesday in May, 1847: and they are hereby requested to make such appointment, and make return thereof to the Secretary of the State Society."

It is customary at each Convention to appoint a member to read a dissertation at the succeeding Convention. On the recent occasion, Thomas Sill, M. D., read a paper entitled, "Observations on Typhus Fever," which is published with the proceedings of the Convention.

Dr. Sill, from his own experience, which appears to have been considerable, thinks that the *typhoid* and *typhus fever* of Louis, Bartlett, and others, are "one and the same disease, differing only according to accidental circumstances, such as the *constitution*, *temperament*, or *habits* of the patient, severity of the attack or malignancy of type."

In reference to the pulse in this disease, the "dissertator" remarks:

" Bartlett and others say, that irregularity of the pulse is rarely found. My experience leads me to differ materially with them on this point; for with two or three exceptions, I have ever found a peculiar irregularity of the pulse, and that early. This irregularity is hardly describable, and yet it is distinct, and, so far as I have observed, peculiar to this form of disease. The pulse usually steadily increases in frequency in the fatal cases, until death, or remains stationary until a decided crisis, and the patient convalesces. And I would here remark, that in a heavy case of typhus fever, no one symptom is better calculated to give increased hope for the patient, than a decrease in the frequency of the pulse." The author is opposed to bleeding and other evacuant remedies, even in the early stage of the disease, and counsels an early resort to tonic and sustaining means.

---

### ASIATIC CHOLERA.

Within the last two or three months, we have had various rumours of the reappearance of the cholera in Asia and the North of Europe, extending over and crossing a portion of the Russian territory. A late number of the London Globe contains the following paragraph in relation to it, which is the latest information on the subject:—

" We have already stated that the cholera had made its appearance in some of the provinces of Persia, carrying death into the principal towns. It has spread from Bokhara to Herat and Meshid, and has now taken the direction from the Caspian Sea to Teheran and Ispahan. Late accounts from Odessa state, that it had crossed the Russian territory and appeared suddenly at Tiflis, taking a northerly direction between the Caspian and Black Sea. On the other side the cholera broke out unexpectedly at Orenbourg, in the mines of the Ural mountains; it crossed the Volga, and set its foot in Europe, at Cassan, only 2000 kilometres from St. Petersburgh. If the accounts we have received are exact, it has taken a most irregular direction. It has advanced from west to north, and does not seem to have followed the banks of the river, as in 1828 and '32. The cholera which devastated France in '31 and '32 had been raging in Persia seven years, 1823 to '30. It first appeared in 1823 at Orenbourg, and shed death around that town for five years. It reappeared at Orenbourg in 1829, and one tenth of the population fell victims. It broke out at St. Petersburgh in July, 1831; and in France in October of the same year."

